# Predictors of explicit and implicit anthropomorphism in house facades

**DOI:** 10.1038/s41598-024-77839-z

**Published:** 2024-11-16

**Authors:** Sandra Weber, Kirsten Kaya Roessler, Kevin Riebandt, Simone Kühn

**Affiliations:** 1 Department of Psychiatry and Psychotherapy, University Medical Center Hamburg-Eppendorf, AG Neuronale Plastizität, Gebäude W37, Martinistr. 52, 20246 Hamburg, Germany; 2 Department of Psychology, University of Southern Denmark, Campusvej 55, Odense M, DK 5230 Denmark; 3 Center for Environmental Neuroscience, Max Planck Institute for Human Development, Lentzeallee 94, D-14195 Berlin, Germany

**Keywords:** House facades, Global Vectors for Word Representation, Architectural psychology, Environmental psychology, Anthropomorphism, Psychology, Environmental sciences

## Abstract

Anthropomorphism describes the tendency to endow objects with human characteristics, with some individuals being more inclined to do this than others. In an ambiguous environment, this phenomenon can offer guidance. This study investigates the relationship between self-reported attribution and evoked anthropomorphism when viewing house facades. Data was collected from three countries (Germany, Denmark, Canada; *N* = 305). Implicit house anthropomorphism was measured using the Global Vectors for Word Representation method. Explicit anthropomorphism was assessed using the Individual Differences in Anthropomorphism Questionnaire (IDAQ) and a specific House Anthropomorphism Score (EHAS). No significant relationship was found between implicit and explicit house anthropomorphism. Individual IDAQ scores were significantly associated with EHAS across all participants, regardless of country. Additionally, a high degree of agreement in explicit ratings between countries suggests that cultural differences are rather negligible. When objects are given human personality traits and people interact with them because emotions are triggered, it is important to understand which aspects elicit positive and reactive behaviors. In particular, houses, which have high psychological significance as objects of self-expression, might contribute to well-being, so research in this area can provide important knowledge for urban design and architecture.

**Supplementary Information:**

The online version contains supplementary material available at 10.1038/s41598-024-77839-z.

## Introduction

The ability of the human viewer to attribute objects and non-human items with emotions or human traits is called *anthropomorphism*; understood as the tendency to fill a behavior into a non-human thing^1^. Humans tend to anthropomorphize as it elicits a feeling of predictability in assuming that the environment is human-like^2^. This form of reasoning is used when a non-human object activates knowledge about humans to make sense of the object^3^. In doing so, the cognitive process of anthropomorphizing might be similar to inductive reasoning, in which existing knowledge is retrieved, supplemented, and applied to the current situation. In this process, the object is interpreted within a familiar frame of reference, which consists of knowledge about people and their behavior^1^. Similarly, as a consequence of anthropomorphism, moral rights are granted to non-human objects, so that meaningful action occurs in the actor, for example by granting a window view to a cuddly toy^4^.

Anthropomorphism as a phenomenon has a long tradition, stemming from religion (e.g. God as human), literature (e.g., talking animals in fairy tales), or art (e.g. the illustrations of Sir John Tenniel for Alice´s Adventures in Wonderland which include a rabbit carrying a pocket watch). In architecture, the human expression has been understood as an important element as well. Already in 1886, the Swiss art professor Heinrich Wölfflin described forms in architecture, which remind us of the human body and therefore provoke certain impressions. Following Wölfflin, a building could, through height, extension, or breadth, provoke the impression of being important, powerful, or sombre^5^.

Research has shown that people do not anthropomorphize in the same way, but that there are interindividual differences in the tendency to do so which are associated with psychological determinants such as social needs or motivation^1^. The current literature on who is more prone to anthropomorphizing is limited. Some studies suggest that older individuals, due to an increased need for social connection, are more likely to anthropomorphize, especially if they are lonely^6^. However, other research indicates that younger, single, creative, and conscientious people, particularly those with a strong relational ability to animals, are also highly susceptible to anthropomorphizing^7^. Complementary neurobiological research illustrated that participants exhibit face-related brain activity when viewing car fronts described with human-like attributes^8^. In architecture, however, discussions often focus solely on architectural styles rather than exploring anthropomorphic or metaphorical interpretations of buildings^9^.

Early research by the psychologists Mary Gauvin and Irwin Altman in the 1980s investigated the social psychological features of homes, highlighting their capacity to reflect cultural and environmental influences^10^. By looking at sites, entrances, and interiors, they show a systematic connection between housing and culture. Their work established a framework for analyzing homes based on two dimensions: identity (reflecting connections between residents) and openness versus closeness. This framework suggests that homes can offer valuable insights into intercultural differences and behaviors^11^.

To investigate if houses are anthropomorphized by observers, a Canadian study, the so-called DalHouse Study^12^, asked students to rate edited photographs of 100 houses on different dimensions including facelikeness (next to typicality and likeability). To investigate how participants associate attributes with houses, we repeated this study using 50 of the 100 houses but added more rating dimensions. The additional rating dimensions—familiarity, freedom, and emotional aspects—were chosen to provide a more nuanced understanding of how participants associate attributes with houses. These dimensions were selected based on their relevance to psychological and emotional responses that can influence anthropomorphic perceptions^13^. Familiarity reflects the psychological comfort of recognizing a place, spatial openness pertains to the perceived freedom or restriction within a space, and emotional aspects like safety or threat relate to fundamental human reactions to spaces. These dimensions align closely with cognitive and emotional processes that underlie how humans anthropomorphize, offering a richer and more nuanced understanding of their responses.

Thus, we obtained ratings with 12 statements for each house. In particular, we were interested in whether our participants rated the same houses highly on the three DalHouse dimensions, as this set of house photos had been thoroughly validated.

Similarly, we aimed to explore possible cultural differences in the rating of the houses, by conducting the study in three countries: Germany, Denmark, and Canada (for more information, see Roessler et al.)^14^. These countries were chosen to represent diverse cultural backgrounds and provide insights into how cultural contexts shape the perception of architectural features. Our study bridges the gap between psychological and architectural assessments, examining how cultural contexts influence anthropomorphic perceptions of house facades. Specifically, we investigated the relationship between self-reported attribution and evoked anthropomorphism when viewing photos of (Canadian) house facades. Understanding the emotional and psychological reactions to buildings, particularly houses, can inform architectural design and urban planning, offering insights into how spaces foster feelings of safety or connection. This is particularly relevant in intercultural contexts, where architectural features may be perceived differently. To address the gap in understanding how houses are anthropomorphized, our study aimed to answer the following three questions: Firstly, how do participants rate houses on dimensions of facelikeness, familiarity, freedom, and emotional aspects (e.g., safety versus threat)? Secondly, are there significant cultural differences in these ratings among the participants from Germany, Denmark and Canada? We expected cultural differences, particularly between Germany and Denmark, based on previous research suggesting variations in cultural perceptions and emotional responses to architectural features^15^. Thirdly, we expected that the degree of anthropomorphism, as implicitly revealed through adjective ratings, can be predicted by explicit measures^16^.

## Materials and methods

### Participants

For this study, participants from three countries (Germany, Denmark, Canada) were recruited via the international online platform Prolific (http://prolific.co). Inclusion criteria included permanent residence in the respective country and native language or fluent language skills in the respective country. 305 individuals (Germany: *n* = 105, mean age = 29.6 years, SD = 9.7, age range: 18–68, 47.6% females; Denmark *n* = 100; mean age = 27.8 years, SD = 8.7, age range: 18–61, 48% females; Canada *n* = 100, mean age = 32.3 years, SD = 11.2; age range: 18–69, 37% females) were invited to take part in this study.

### Ethical considerations

The study was approved by the local ethics committees of the University Medical Center Hamburg-Eppendorf, Germany (LPEK-0269) and the Danish Research and Innovation Organization (RIO) of the University of Southern Denmark (no.11.298). The experiment was performed in accordance with the relevant regulations and guidelines.

### Procedure

The questionnaire was implemented in Inquisit 6 (Millisecond, 2021). Participants were asked to read the study information and agree if they meet the eligibility criteria. Afterwards, they gave their informed consent to participate in the study. Subsequently, participants were given an identification number and the link to the study via prolific. The Danish and Canadian participants answered the original English version; the German participants received a professionally translated version in German. The questionnaire consisted of three different phases: A) Implicit measure (implicit house anthropomorphism score, IHAS): At the beginning of the study, the participants were exposed to the photographs of house facades. For each facade, they were asked which adjectives spontaneously came to their mind. They completed the sentence “This house is….” and “This house makes me….“. Example answers to these questions are: “active”, “lonely”, “unattractive”.

- B) Explicit measure (explicit house anthropomorphism score, EHAS): Following the implicit measure, participants were asked to answer 12 statements about each facade using a slider ranging from 0 = “not applicable at all” to 100 = “very applicable”. The 12 statements were: (1) The house has a face (facelikeness). (2) This house is a typical house (typicality). (3) This kind of house looks familiar (familiarity). (4) This house looks childlike (childlikeness). (5) This house looks inviting (invitingness). (6) This house looks friendly (friendliness). (7) I like this house (liking). (8) I would feel safe in this house (safety). (9) This house looks threatening (threat). (10) In this house I would feel lonely (loneliness). (11) In this house I would feel free (freedom). (12) I would like to live with others in this house (with others).
- C) General anthropomorphism measure: At the end of the study, participants answered the nine items of the short version of the Individual Differences in Anthropomorphism Questionnaire (IDAQ). The IDAQ is a psychometric measure to quantify individual differences in the expression of anthropomorphism^4^. Two example items are: “To what extent does the average robot have consciousness?” and “To what extent does a tree have a mind of its own?” The internal consistency of the IDAQ is often indicated by Cronbach’s alpha, which ranges between 0.70 and 0.88. In our study, measures for internal consistency were: across all three groups, Cronbach’s alpha was α = 0.73. For the individual groups, it was α = 0.67 for Germany, α = 0.75 for Canada, and α = 0.76 for Denmark. With the exception of Germany, these values correspond to the standard values.

We used this test instrument to examine whether subjects with a higher expression of anthropomorphism tend to use more human adjectives to describe houses.

Finally, subjects provided demographic information. To fill in the questionnaire lasted between 75 and 90 min. The participants received approximately 11 euros as an expense allowance when having completed the questionnaire.

### Stimulus material

**Fig. 1:**
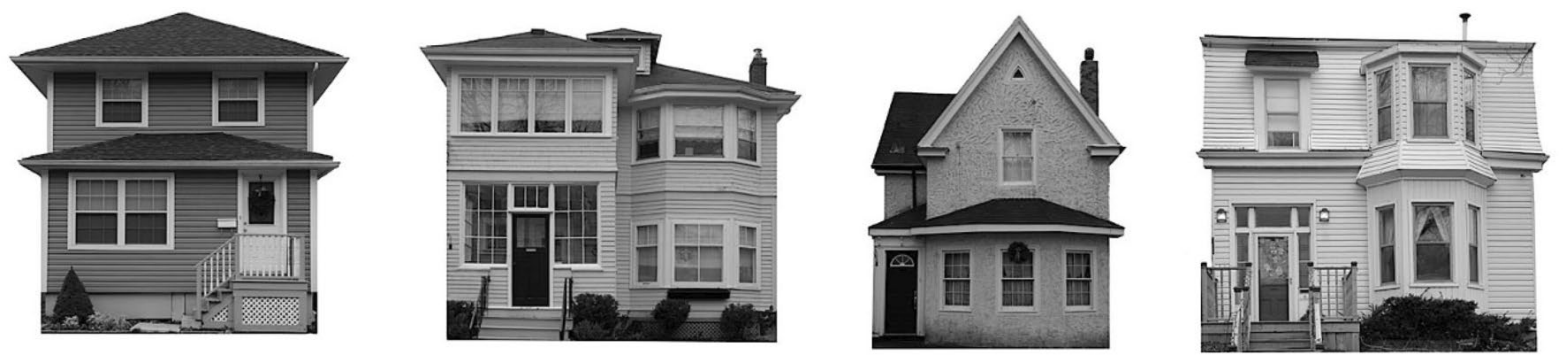
Example photos from the DalHouse^12^set (see 10.6084/m9.figshare.1279430.v6 for original pictures in database). As stimulus material, we used 50 house facades (see Fig. 1) from the so-called “DalHouses” (Filliter et al., 2016). The original set contains 100 Canadian houses, which have previously been used to study typicality, familiarity, and degree of similarity to faces.

The stimulus material consisted of 50 house facades (see Fig. 1) from the so-called “DalHouses”^12^. The original set contains 100 Canadian houses, which have previously been used to study typicality, familiarity, and degree of similarity to faces. To limit the time of the study, we sorted the DalHouse house facades according to the average face judgment and selected every other house facade for our study to reach a broad distribution across the facelikeness dimension^14^.

### Data analysis

Data was preprocessed and analyzed descriptively using SPSS version 29. Inference statistical analyses were performed using the R programming language version 4.2.2 (r-project 2022). The hierarchical linear models were performed with the LME4 package, version 1.1–30.

One of the questions to be investigated was whether a house’s implicit humanness rating can be predicted from four statements on which a participant could explicitly rate whether a house had a particular characteristic (facelikeness, childlikeness, friendliness, threat). These four statements together form the explicit house anthropomorphism score (EHAS). Since the humanness of the houses was explored by means of adjectives about the completion of the sentences above, a method of quantification had to be developed. For this implicit house anthropomorphism, the method of the Global Vectors for Word Representation algorithm was applied^17^. This is an unsupervised learning algorithm for obtaining word vectors, which can extract semantic information from long texts. The basic idea is grounded on the assumption that the meaning of a word is largely determined by the words with which it frequently co-occurs. For this, the model uses a large amount of text data, calculates the probability of words co-occurring, and thus includes information about the associations between the words. Each word is represented as a vector in a dimensional space, with the position of these vectors reflecting the relationships between the words. In the GloVe method, training was based on aggregated global correlations concerning words that are often clustered in the same context. The training procedure aims to adjust the vectors to reflect logarithms of the probabilities of word co-occurrences. For this, a cost function was used to minimize the differences between the scalar products of the word vectors and to approximate the actual probabilities of co-occurrence. This approach enables GloVe to capture semantic relationships between words. Similar words that occur in similar contexts are positioned close to each other in this vector space. This allows the model to recognize semantic analogies.

For this study, a pre-trained model was used, which is as close to colloquial language as possible and considers as many linguistic facets and contents as possible. We chose a model trained on the contents of Wikipedia as of 2014, as well as the “English Gigaword, 5th edition,” a comprehensive archive of newswire text data (for a detailed overview, the mentioned source can be consulted)^18^. In our analyses, we assume that if the average value of words perceived as human and the adjective mentioned by the subject lie close together in vector space, i.e., the distance between the vectors is small, then the mentioned adjective can also be considered human. If this is not the case, it can be assumed that the adjective is not perceived as human. This distance between the vectors was calculated using the Euclidean distance.

Subsequently, all adjectives used by the subjects in the study were quantified in terms of their “humanness” to build an implicit house anthropomorphism score (IHAS)”. The specific procedure, in this case, was as follows: From the open-ended responses, all adjectives were filtered out and duplicates were removed. Those adjectives that were mentioned in German were translated into English using five different online translation programs (deepL, promt.one, pons, bing, google translator; for the Danish translations we used deepl, bing, google, babla und ordbog.dk). The most frequent translation result was selected as the final translation for further processing. We assumed that adjectives that frequently co-occur with words and that have a high content relatedness to humanness also indicate that the corresponding house was perceived as “human”. We created a list with particularly “human” words (see Table 1). This list was evaluated independently by nine psychologists from our research group on an 11-point scale ranging from 0 = no relation to humanness at all to 10 = very strong relation to humanness. We used Kenndall´s W to estimate interrater reliability with a resulting estimate of 0.91. There was no need to exclude any words.

Subsequently, each adjective entered by the participants in response to the presented houses was tested for its degree of semantic closeness to the human-related words. We assume that if the semantic proximity to the above human words list is high, on average, the adjective used by the participants should also be classified as human, and thus the house was implicitly perceived as human.

**Table 1:** Human words.

Acquaintance	Friend	He	Man	People	Student
Boy	Girl	Hero	Member	Person	Team
Child	Girlfriend	Human	Men	Pupil	They
Employee	Grandfather	Husband	Neighbor	Relative	Wife
Enemy	Grandmother	Idol	Opponent	She	Woman
Family	Granny	Kid	Partner	Siblings	Worker

Listed are all human nouns that have been entered into GLoVE.

For the calculation of the semantic proximity of the individual adjectives to the above words, the calculation of a distance metric, here the cosine distance, was used. Distance metrics are generally used whenever the similarity of points in space is to be determined and compared^19^. For theoretical reasons, the cosine distance is regularly used for word vectors. Cosine similarity is scale-invariant and considers the directional relationship between vectors, which is crucial in natural language processing. This makes the cosine distance less sensitive to fluctuations in the number of occurrences of terms that are semantically similar. The smaller the angle, the greater the similarity. However, since we wanted to be exhaustive and explore the stability of the results, we also calculated other distance metrics such as Euclidean distance. The calculation was done in the form of the median as well as the mean value in each case. However, a correlation analysis revealed that all variants we calculated demonstrate high intercorrelations so the following analyses were conducted only with the cosine distance, as this seemed theoretically advisable (correlations see Appendix A, Table A1). The value range of the cosine distance in our case was between 0 and 2, where 0 indicates an extremely high semantic closeness and 2 an extremely low semantic closeness.

As a result, we obtained an evaluation of how “human” this word is with respect to the model using the distance measure for each adjective, that participants entered in the free text response box in part A) of the experiment. Since the participants usually indicated several adjectives per house, we calculated the average value of the cosine distance for all adjectives used per house. This value indicates how humanly a house was rated by a given participant.

As described above, our hypothesis was that the extent of humanness calculated based on the adjectives (implicit house anthropomorphism score, IHAS), can be predicted by the four items of phase B) (explicit house anthropomorphism score, EHAS) as well as the IDAQ of phase C) (general anthropomorphism measurement) of the experiment.

To account for the fact that each participant is likely to rate the houses more similarly than would be expected between participants, a hierarchical linear model (HLM) was used to examine the relationship between the four items that ask about human characteristics 1 (facelikeness), 4 (childlikeness), 6 (friendliness), and 9 (threat) (EHAS) in relation to houses and the IHAS with crossed random factors for participant ID and image number. Then a linear regression was calculated for the association of IDAQ with the IHAS. Furthermore, two ANOVAs were calculated to determine the influence of nationality on the IHAS derived from the adjectives and the IDAQ mean score, respectively. We used regression analysis to reveal the association with age and the IDAQ.

## Results

### Implicit house anthropomorphism score (IHAS)

There was no significant relationship between any of the 4 items that were used to build the EHAS (see Appendix B, Table B1). Furthermore, there was no effect regarding participants’ nationality on IHAS. This was confirmed by the analysis of variance, which showed no significant mean difference between nationalities with regard to the IHAS (F(2,303) = 2.121, *p* = .122, ηp2 = 0.014 ). However, there was an indication for a significant age effect on how human a house was evaluated by the participants using adjectives.

### 12-statement-rating (explicit measurement)

The evaluation of the 12 statement questions showed that for seven questions (1, 5, 7, 8, 9, 11, 12), subjects from Germany, Canada, and Denmark each rated the same houses highest (see Tables 2 and 3). Furthermore, for the three questions (1,2 and 7) used for the DalHouse study^12^, the same three houses were rated highest in our study as well (see Table 4). House No. 31 was rated most unequivocally and across countries, when it came to the question of facial similarity (> 25,000 points out of a possible 30,500 points).

**Table 2:** Results of the 12-statement-rating.

Statement		Houses with the highest score							
		Overall (*n* = 305)		Germany (*n* = 105)		Canada (*n* = 100)		Denmark (*n* = 100)	
No		House No	Score	House No	Score	House No	Score	House No	Score
1	Facelikeness	31	**25,258**	31	**8399**	31	**8106**	31	**8753**
2	Typicality	45	20,238	8	6906	45	7169	8	6840
3	Familiarity	45	19,859	8	6896	45	7213	44	5992
4	Childlikeness	32	15,797	32	5486	32	5274	31	5473
5	Invitingness	46	21,304	46	7186	46	7100	46	7018
6	Friendliness	46	20,650	44	7157	45	7003	15	7083
7	Liking	46	22,911	46	7781	46	**7732**	46	7398
8	Safty	46	**23,116**	46	**7963**	46	7573	46	**7580**
9	Threat	39	19,514	39	6471	39	6641	39	6402
10	Loneliness	34	17,963	4	6155	39	5881	34	6345
11	Freedom	46	21,340	46	7353	46	6869	46	7118
12	With others	46	**23,698**	46	**7846**	46	**7836**	46	**8016**

Each participant (*n* = 305) rated 50 houses with 12 statements using a slider ranging from 0 = “not applicable at all” to 100 = “very applicable”, with the highest possible result of 30,500 points for one statement. The values in bold indicate the values that scored the highest overall and within each country. With one exception, the results were the same in all three countries.

**Table 3:**
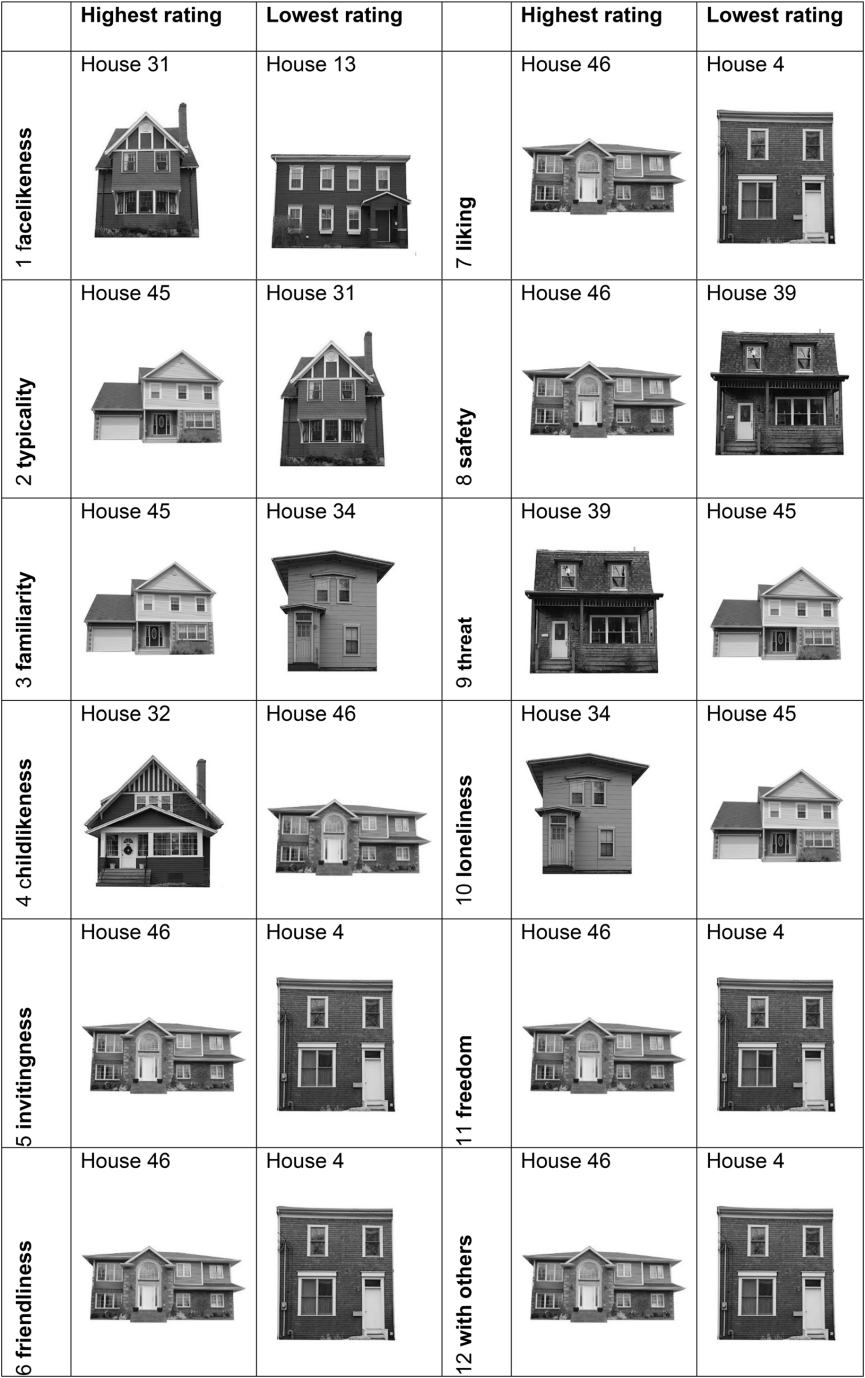
Results of the 12-statement-rating (overall) in pictures^12^.

**Table 4:**
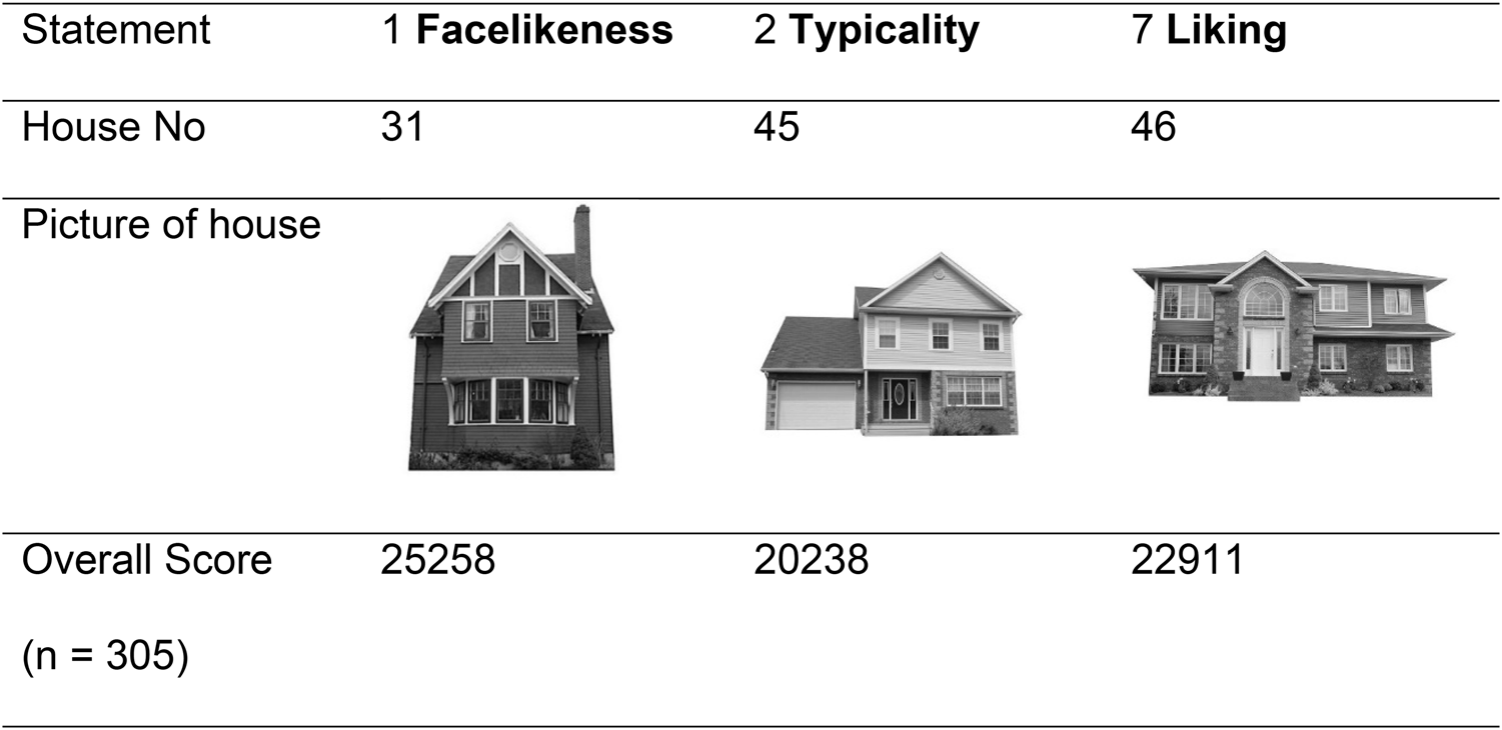
Replication of the DalHouse-rating.

The three statements are the ones used identically in the DalHouse-rating^12^. The same houses received the highest ratings in our study as in the DalHouse-rating.

### General anthropomorphism measurement (IDAQ)

**Fig. 2:**
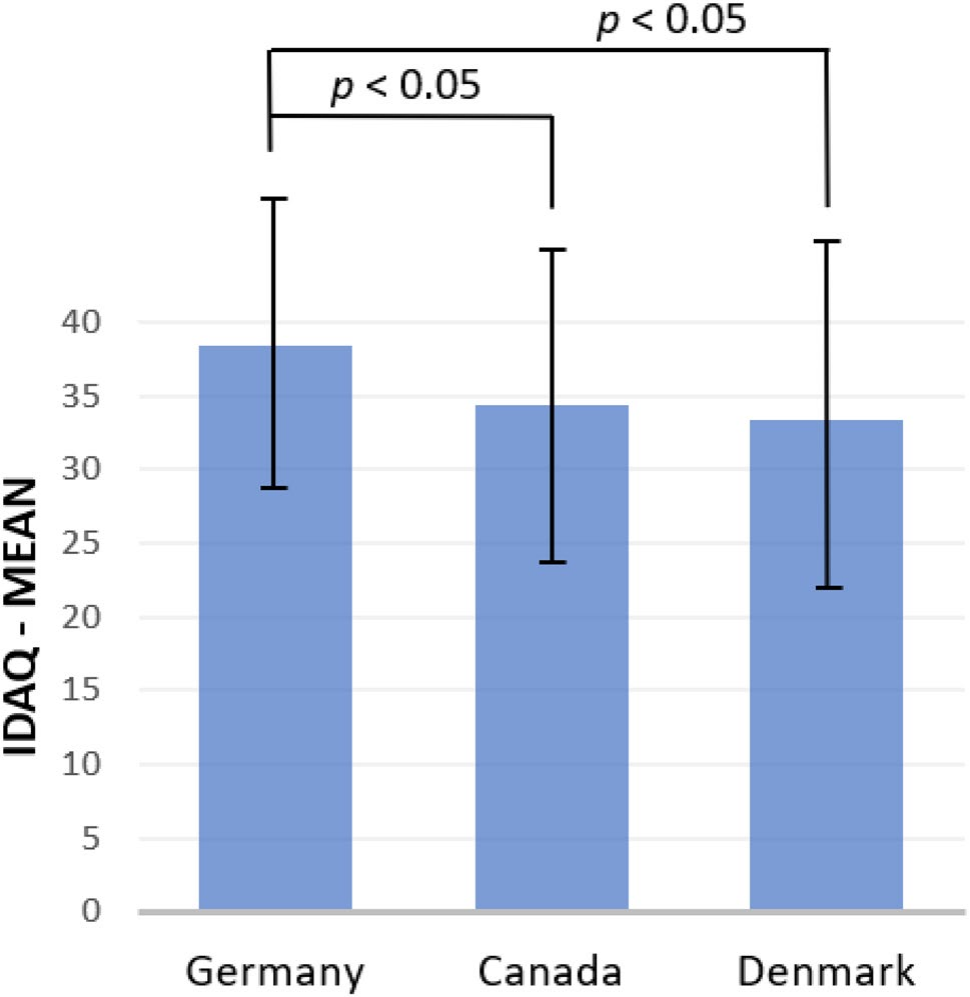
Bar chart with mean values of the IDAQ sum score by country. The figure shows graphically for which countries there is a significant mean difference in the IDAQ.

Nationality was predictive of participants’ mean score in the IDAQ (F(2,302) = 5.895, *p* = .003, ηp2 = 0.038). The IDAQ^4^ revealed that the tendency of subjects to anthropomorphize was significantly different between German and Danish participants as well as between German and Canadian participants (see Table 5; Fig. 2). The German participants anthropomorphized more than the others, whereas there was no difference between Danish and Canadian participants. However, the linear regression designed to calculate the relationship between the IDAQ and the IHAS did not yield a significant difference (F(1,303) = 0.772, *p* = .380, f = 0.054).

**Table 5:** Results of the IDAQ.

Country	*n*	MD	SD
Germany	105	38.42	10.36
Canada	100	34.30	11.58
Denmark	100	33.30	12.09

The highest score achievable on the short version with 9 items of the IDAQ is 90.

The following figure (Fig. 2) shows for which countries there is a significant mean difference in the IDAQ:

### Explicit house anthropomorphism score (EHAS)

The regression analysis revealed that each year of life was associated with a significant reduction of the EHAS by -46.37 points (*p* = < 0.001, 95% CI -65.43 to -27.31). That is, the older our subjects were, the less anthropomorphic they responded to the questionnaire concerning the houses. Likewise, the EHAS was positively associated with the IDAQ scores. Each IDAQ point was significantly associated with an increase in the EHAS by 17.37 points (*p* = .041, 95% CI 0.75 to 34), i.e., the higher the expression of anthropomorphism in the subjects (as measured by the IDAQ), the more human characteristics they conferred on the houses in the EHAS.

## Discussion

Our main aim was to investigate the relationship between a general measure of anthropomorphism and explicit as well as implicit anthropomorphism of individuals when viewing photos of typical Canadian house facades. As stimuli, we used photos from the DalHouse study, and expanded with additional answer categories^12^.

As stated above, the literature investigating which individuals tend to be more prone to anthropomorphize is not sufficient. The results of our study suggest that younger, single, more creative, and more conscientious people are particularly prone to anthropomorphism. However, it is important to note that our sample was relatively young (M = 29.88, SD = 10.06) and that only a total of 17 subjects were 50 years of age or older. While our age group was limited by practical considerations resulting in a relatively young sample, we acknowledge the potential impact of age as a covariate.

Contrary to our assumption, subjects who showed a tendency to anthropomorphize in the IDAQ did not use human adjectives more often (IHAS) to describe the houses shown. Hence, we were not able to predict, based on the individual IDAQ score, to what extent subjects would anthropomorphize the houses in the descriptive open-ended question. However, when we picked the four questions from the 12 statement questions clearly related to explicit anthropomorphism and combined them into a so-called EHAS, these scores correlated significantly with the, also explicit, IDAQ scores. Thus, the individual total score of the IDAQ predicted the respondents’ tendency to reply to these four selected explicit questions, regardless of nationality. In other words, it was possible to use one explicit measure (IDAQ score) to predict another explicit measure (EHAS). This raises the question of whether it is correspondingly possible to use other implicit anthropomorphism measures, such as brain imaging data (e.g., activity in the fusiform face area (FFA)), to predict IHAS-scores. In a study by our group, subjects with an implicit tendency to anthropomorphize car fronts (based on raters who judged the degree to which each adjective that participants generated in a free text applied as a characteristic of human beings) showed a higher activity in the FFA, the area in the brain that is responsible for face processing^8^. Future studies may want to test the association between such an implicit anthropomorphism score or the IHAS and brain activity in FFA during the observation of house facades.

There are some additional explanations of why the IHAS calculated was unrelated to other aspects of the houses to which humanness can be attributed. On the one hand, some of the questionnaires were filled out in the language of the respective country the participants were tested in, which could have led to distortions when translated into English, the language we used as the basis for our analyses.

The cross-national results of the four statements (EHAS) suggest that the tendency to anthropomorphize is independent of culture, at least for the cultures assessed in our study. This conclusion is in line with a literature review from 2016 summarizing 509 online survey respondents: anthropomorphism was found to be related to personality traits, as well as age, and relationship status. In addition, personal relationships with animals and the influence of past experiences also appeared to be important^20^. Similarly, Epley, Waytz, and Cacioppo argued that people in industrialized societies tend to attach human-like terms to animals more than people from rural areas, who interact with farm animals more frequently in their daily lives and therefore might have a more pragmatic knowledge representation of animals^1^. Conversely, rural residents may be more inclined to anthropomorphize modern, mechanical objects (e.g., computers)^1^. This could mean that house types located in an urban environment are also more likely to be anthropomorphized by the rural population and vice versa. However, in the present study, the house photos used, could not be clearly assigned into a rural or an urban category, and we also had no information as to whether the subjects lived in a rural area or in a city.

Although the 50 photos used displayed typical Canadian houses, subjects from three different countries on two continents rated the same houses highest on seven of the 12 statements. The three statements for which the houses received highest ratings asked about similarity to a face, safety, and community living. Again, with one exception, all but one of the three countries ranked the same three houses among the top three. This is all the more surprising as it could be assumed that the subjects, with their different cultural backgrounds, would also display different perceptions of houses. These results could suggest that the house characteristics we tested here are culture-independent, at least for the cultures under research in the present study. But we caution the reader to generalize the results as our selection of countries was limited. All three countries can be categorized as WEIRD (Western, Educated, Industrialized, Rich, and Democratic)^21^, making a more diverse selection of countries and/or cultures necessary.

Why do we consider it relevant to investigate whether and which facades humans anthropomorphize and why do we assume this can influence their behaviour or well-being? First, research showed that human behaviour can be unconsciously influenced by the presentation of words^22^, by the presentation of situations^23^, and by the presentation of other people^24^. Only later did Chartrand find that anthropomorphized objects can elicit effects on the behaviour of subjects^7^. Participants in her study anthropomorphized objects (dogs, cats) and behaved according to the attributed properties without being aware of the objects’ influence^7^. Another study showed that subjects who were previously primed with the Apple logo solved a task more creatively than those who were previously subliminally presented with an IBM logo^25^. Here, participants adopted the characteristics of an anthropomorphized consumer brand. Thus, evidence indicates that anthropomorphized, non-human entities are perceived as possessing features of a human personality and that people interacting with them show behavioural reactions as if their counterparts were human. Accordingly, house fronts could be understood and potentially used as complex social stimuli that evoke certain behaviours. In this way, it would be conceivable to induce reactive behaviours in residents and citizens and thus, for example, increase the general level of well-being in neighbourhoods. This is because one’s home does not only represent a physical space but also has a psychological significance^11^. One’s home contributes to the satisfaction of basic human needs for control and attachment^26,27^, and, at the same time, represents an object of self-expression that is associated to an increase in self-esteem^28^. If houses are anthropomorphized, a social connection to the human-like characteristics of the house could be established. This, in turn, would increase the personal value of the house, as has been described for other products like laptops, cell phones, USB drives, and toothbrushes^29^, or lead to longer-term attachment to the anthropomorphized object^30^.

Accordingly, it seems reasonable for future research to attempt identifying house characteristics that are sources of activation of unconscious human-related constructs in viewers.

### Summary and limitations

Within the scope of the present study, we described a method for processing qualitative data with the aim of classifying descriptive adjectives based on their semantic distances in relation to humanness. Since, to our knowledge, there is no previously evaluated list of such terms, we created a list of nouns that we understand to be frequently used to indicate humans. This could be seen as a strength or a critical point that should be considered in future research.

In addition, in part A) of the study, two qualitative questions were jointly presented namely: “This house is….” and “This house makes me….”. While the first part asked for a descriptive adjective, the second part asked for an emotion. Combining both aspects into one category might pose methodological challenges. E.g., is “happy” meant as a description for a happy house or for becoming happy, when seeing the house. This challenge was approached by collapsing all answers into a single semantic category. While our sample did not aim to be representative of the general population, it was sufficient for exploring our research questions within the scope of this study. The relatively narrow age range may influence the interpretation of the age-related findings, and it remains unclear how the age effect should be interpreted in comparison to other previous studies looking at age, as the age structure of our sample (young and narrowly defined) differs significantly. Including a broader age range in future studies could offer additional insights into the relationship between age and anthropomorphism scores.

We assessed the employment status during data collection, but we did not assess interest in architecture. This should be taken into account in future research, as it could be an influencing factor that should not be underestimated.

We were able to show that in the overall results the same houses as in the DalHouse study were rated highest by our subjects^12^. This is very close to a replication, but our study consisted of only 50 and not 100 houses as in the original study. Therefore, an exact replication cannot be concluded.

## Conclusion

Anthropomorphism is an important variable in understanding the meaning people attach to non-human objects. To investigate the relationship between self-reported attribution and evoked anthropomorphism we used a new method to examine the semantic proximities of adjectives to human words. Although we could not demonstrate a statistical relationship between the general tendency to anthropomorphize and the use of human adjectives in free responses to houses, we found that the IDAQ, as an explicit measure for anthropomorphism, could predict another explicit measure (the EHAS). The developed EHAS consists of only four questions that refer to a specific object (here: house facades), but correlates with the general construct of anthropomorphism of the validated IDAQ (convergent validity). This short form of the EHAS could be used as a test instrument in future research that reliably detects whether a house facade is anthropomorphized. In the long run, this would allow the identification of specific housing factors that enhance the well-being of residents through positive interaction, providing valuable insights for urban design and architecture.

## Electronic supplementary material

Below is the link to the electronic supplementary material.

Supplementary Material 1

## Data Availability

The datasets generated during and/or analysed during the current study are not publicly available due to data security protection but are available from the corresponding author upon reasonable request.
