# Supplementary material for: Predictors of explicit and implicit anthropomorphism in house facades
Source: Sci Rep. 2024 Nov 16;14:28291. doi: 10.1038/s41598-024-77839-z (PMC11569235; doi:10.1038/s41598-024-77839-z)
Supplement: Supplementary file 1 — Supplementary Material 1 [file 41598_2024_77839_MOESM1_ESM.pdf]

Appendix A

As the following table shows, all distance measures correlate highly with each other. The restriction to the measure we have chosen is therefore not only theoretically but also empirically unproblematic.

Table A1 CORRELATIONS BETWEEN THE DIFFERENT SEMANTIC DISTANCE MEASURES

**Distance measure**	*Mean_CS_MD*	*Median_CS_MD*	*Mean_CS_Mean*	*Median_CS_Mean*	*Mean_EC_MD*	*Median_EC_Mean*	*Mean_EC_Mean*	*Median_EC_Mean*
Mean_CS_MD	1	.937**	.996**	.944**	.910**	.867**	.906**	.862**
Median_CS_MD	.937**	1	.929**	.953**	.847**	.868**	.842**	.861**
Mean_CS_Mean	.996**	.929**	1	.946**	.910**	.866**	.907**	.861**
Median_CS_Mean	.944**	.953**	.946**	1	.854**	.862**	.849**	.856**
Mean_EC_MD	.910**	.847**	.910**	.854**	1	.919**	.996**	.915**
Median_EC_MD	.867**	.868**	.866**	.862**	.919**	1	.913**	.983**
Mean_EC_Mean	.906**	.842**	.907**	.849**	.996**	.913**	1	.917**
Median_EC_Mean	.862**	.861**	.861**	.856**	.915**	.983**	.917**	1

Note: cosine distance (CS), euclidean distance (EC) ** The correlation is significant at the 0.01 level (2-sided)

Appendix B

Table B1 UNSTANDARDIZED LOADINGS OF THE HIERARCHICAL LINEAR MODEL (HLM) IN RELATION TO IHAS

**Variable name**	*Estimate*	*Std. Error*	*P-Value*	$Q_{0.025}$	$Q_{0.075}$
intercept	7.12E-01	1.41E-02	2.00E-16	6.84E-01	7.39E-01
facelikeness	-2.92E-05	3.90E-05	0.4541	-1.06E-04	4.74E-05
childlikeness	4.45E-05	4.42E-05	0.3146	-4.22E-05	1.31E-04
friendliness	-5.58E-05	5.48E-05	0.3088	-1.63E-04	5.16E-05
threat	-6.76E-06	5.28E-05	0.8981	-1.10E-04	9.68E-05
age	5.09E-04	2.54E-04	0.0461*	9.43E-06	1.01E-03
sex	-1.03E-03	4.86E-03	0.8328	-1.06E-02	8.52E-03
nationality	-4.03E-03	3.09E-03	0.1933	-1.01E-02	2.05E-03

Note: * The correlation is significant at the 0.05 level (2-sided)
